# Antibiotic prescription for older patients in end-of-life care: a randomized survey among physicians in Switzerland

**DOI:** 10.1186/s12877-026-07477-9

**Published:** 2026-05-12

**Authors:** L. Papasokrati, S. Pautex, F. Herrmann, P. Vayne-Bossert, V. Prendki

**Affiliations:** 1 Department of Rehabilitation and Geriatrics, Geneva University Hospitals, Geneva, Switzerland; 2 Department of Rehabilitation and Geriatrics, Division of Palliative Medicine, Geneva University Hospitals, Geneva, Switzerland; 3 Department of Medicine, Division of Infectious Diseases, Geneva University Hospitals, Geneva, Switzerland

**Keywords:** End of life, Aspiration pneumonia, Antibiotics, Geriatrics, Palliative care

## Abstract

**Introduction:**

Pneumonia is a common infection at the end of life, yet the benefits of antibiotics for symptom relief or prolonged survival remain uncertain. This study aimed to investigate physicians’ attitudes and factors influencing antibiotic prescription in aspiration pneumonia in frail older patients in palliative care settings across Switzerland.

**Methods:**

A survey was conducted among physicians practicing in internal medicine, geriatrics and family medicine, in hospital or ambulatory settings across Switzerland’s three linguistic regions. Participants were randomized to receive one of three clinical vignettes describing a geriatric patient with aspiration pneumonia, with variations in advance directives, functional dependency and dementia status. They were asked whether they would prescribe antibiotics and to explain their reasoning. The survey also collected participant demographics, including age, linguistic region, practice setting and palliative care experience.

**Results:**

Among the 195 participants, 22.1% opted to prescribe antibiotics. The primary reason for prescribing antibiotics was to alleviate dyspnea. Physicians were significantly more likely to prescribe antibiotics for patients with better functional status. Notably, 76.4% believed withholding antibiotics could shorten life, while 77.9% did not think antibiotics improved comfort. Physicians practicing in French-speaking regions were more likely to prescribe antibiotics than their counterparts in German- and Italian-speaking regions (33.7% vs 13.8% and 11.1% respectively.) Age, practice setting and palliative care experience were not significantly associated with prescribing behaviour. Additionally, 77.9% of physicians stated that they would modify their decision if the patient’s family disagreed with their initial choice.

**Conclusion:**

These findings highlight ongoing uncertainties and cultural variations in antibiotic prescribing practices at the end of life, highlighting the need for clinical decision-support tools and educational resources directed not only at physicians, but also at nursing teams, families, and caregivers. In the context of an aging population and rising antimicrobial resistance, a deeper understanding of the factors influencing antibiotic use in palliative care is essential to inform the development of targeted antimicrobial stewardship interventions aligned with patient values and palliative care goals.

**Supplementary Information:**

The online version contains supplementary material available at 10.1186/s12877-026-07477-9.

## Background

Infections are common among older patients in end-of-life care—either as a direct consequence of the underlying disease or due to ageing, immunosenescence or immunosuppressive treatments—and frequently represent the proximate cause of death [31, 36]. The use of antibiotics in end-of-life care presents a complex clinical and ethical challenge, compounded by the presence of very few evidence-based consensus guidelines [2].

Observational data indicate a substantial use of antibiotics in this population, across both inpatient and outpatient settings [13, 26]. Antibiotics are frequently the last medications to be discontinued and are often maintained during the final days of life [4, 22] primarily with the intent to provide symptom relief and enhance patient comfort [6, 12].

However, the therapeutic benefit of antibiotics in this context remains uncertain. With the exception of symptomatic lower urinary tract infections (and even then, the evidence is still weak), available evidence suggests that antibiotics provide limited symptom relief while posing significant risks, including gastrointestinal adverse effects, *Clostridioides difficile* infections, and discomfort associated with invasive administration routes such as intravenous lines in addition to other possible side effects [32]. In a study involving long-term care facility residents with advanced dementia, antibiotic treatment was associated with greater levels of discomfort compared to no treatment [18]. Furthermore, antibiotic use has been linked to prolonged hospital stays [9] and does not appear to confer meaningful survival benefits [35].

Considering the increasing concern regarding antimicrobial resistance, recent stewardship recommendations advocate for a cautious and judicious approach. Antibiotic use at the end of life should be considered a potentially aggressive intervention, warranting a careful risk–benefit assessment. In addition, expert opinion guidelines emphasize the importance of clearly defined therapeutic goals and the integration of antibiotic-related decisions into advance care planning [1, 5, 16].

Despite these recommendations, little is known about how physicians’ attitudes and decisions vary when confronted with frail older patients at the end of life, particularly across different cultural and linguistic regions of Switzerland. Understanding these variations could help identify the factors that shape clinical reasoning and highlight potential targets for stewardship interventions.

This study therefore aimed to describe physicians’ attitudes and factors influencing antibiotic prescription for older patients with aspiration pneumonia at the end of life. Using randomized clinical vignettes varying in functional status, cognitive impairment, and advance directives, we explored how these patient characteristics, together with physician attributes, were associated with reported antibiotic prescribing.

We expected that physicians would report a higher likelihood of antibiotic prescription for patients with better functional status, and a lower likelihood when severe dementia or advance directives were present. We further anticipated regional differences, with higher prescribing in French-speaking areas.

## Methods

### Study design

A multicenter survey study was conducted in which participants were randomly assigned to receive one of three clinical vignettes that varied according to the presence of advance directives, the level of functional dependency and the dementia status of the patient described.

Participation was voluntary and no incentives were offered. Data was collected between September 2024 and October 2024 using the REDCap platform hosted on the secure servers of Geneva University Hospitals. Each participant received a unique survey link, preventing multiple submissions. Participants had the choice to complete the survey in one of the three following languages: French, German or Italian. Only fully completed questionnaires were analyzed.

### Survey development

An artificial intelligence translation software (“DeepL Translator”) was employed for the translation from French to Italian and German, followed by verification and correction by Italian-speaking and German-speaking physicians. The questionnaire was then submitted to five internal medicine physicians for comprehension testing.

### Population

The target population consisted of physicians who had completed their training in general internal medicine, family medicine and/or geriatrics and were practicing in outpatient settings or hospitals across the three linguistic regions of Switzerland (French-, German- and Italian-speaking parts). In total, we contacted 1912 outpatient physicians and 543 hospital physicians.

Physicians practicing in outpatient settings were contacted by conventional post and received a letter of information alongside a QR code providing access to the online questionnaire. Their addresses were obtained from the SASIS database (the umbrella association of Swiss health insurers) for the purpose of the study.

Physicians practicing in hospital settings were contacted via email, containing a link and a QR code. Their email addresses were obtained with the permission of each department’s head physician.

All participants were randomized to receive one of the three versions of the questionnaire containing a slightly different clinical vignette.

Participants were initially given a deadline of three weeks to answer the questionnaire. A reminder was sent at the end of the three weeks with an additional two-week deadline for those who had not yet replied. Participants were only able to complete the questionnaire once.

### Clinical vignettes

The clinical vignette at the beginning of the survey presented the case of an 82-year-old female patient with significant comorbidities, hospitalized for aspiration pneumonia, treated with piperacillin and tazobactam with initial improvement. Later, she presented with new-onset dyspnea, fever, and delirium, hinting towards a new episode of aspiration pneumonia.

Three slightly different versions of the vignette were created, with differences regarding functional status, the presence or absence of dementia, as well as the presence or absence of advance directives. The patient with moderate functional dependency lived at home with her husband, while the other two patients with major functional dependency lived in long-term care facilities. All other aspects of the vignettes were identical. Table 1 summarizes the differences between the three vignettes. The complete clinical vignettes can be found in the appendix.

**Table 1:** Summary of the differences between the three vignettes

	**Cognitive impairment**	**Degree of functional dependency**	**Advance Directives**
Vignette 1	Present	Severe	Absent
Vignette 2	Absent	Severe	Present
Vignette 3	Present	Moderate	Absent

### Randomization

To ensure balanced allocation of vignettes across participants’ characteristics, we used stratified randomization based on practice setting (hospital versus ambulatory practice) and linguistic region (French-speaking versus German-speaking versus Italian-speaking).

### Survey questionnaire

The survey included 12 items addressing the clinical vignette and 8 items related to participant demographics. It included four types of questions: (i) yes/no items exploring prescribing attitudes and decision-sharing; (ii) two 0–10 rating-scale items assessing how strongly various clinical factors influenced the decision to prescribe or withhold antibiotics; (iii) a multiple-choice item on the perceived objectives of antibiotic treatment; and (iv) demographic questions on age, practice region, years of experience, practice setting, and palliative care training.

Participants were first asked whether they would prescribe antibiotics for the patient described in the vignette. Subsequent questions explored the rationale behind their decision, the intended goals of treatment, their willingness to discuss the decision with the care team and/or the patient’s family, and their views on the appropriate role of antibiotics in end-of-life care. Participants were also asked to indicate their level of certainty regarding their decision. Demographic variables were collected at the end of the questionnaire.

### Statistical analysis

Statistical analyses were performed using two-sided Fisher’s exact tests for dichotomous categorical variables (yes/no questions). Differences in antibiotic prescription between the three vignette versions were assessed using one way analysis of variance by ranks (Kruskal–Wallis test). For rating scale questions, analysis of variance was applied, with Bartlett’s test to assess homogeneity of variances and Bonferroni’s post hoc test to evaluate pairwise differences between vignettes when applicable. For demographic questions containing categorical variables (age, years since medical degree, linguistic region), the Kruskal–Wallis test was applied. P was defined as statistically significant when < 0.05. All statistics were conducted with Stata, release 18.0.

## Results

### Participant demographics

Of the 2,455 physicians invited to participate, 195 fully completed the questionnaire, yielding a response rate of 7.9%. Among the 195 respondents, 44.6% worked in private practice, 45.1% in hospital settings, and 10.3% practiced in both. Among hospital-based physicians, 48% worked in general internal medicine wards, 50% in geriatric wards, and 2% in hospital-based primary care.

Regarding linguistic regions, 42.6% of participants practiced in French-speaking areas of Switzerland, 48.2% in German-speaking regions, and 9.2% in Italian-speaking regions.

Previous exposure to palliative care varied considerably: 40.5% of participants reported no prior experience, while 34.9% had more than one year of experience. Only 14.4% held a formal qualification (degree or certificate) in palliative care.

### Overall antibiotic prescription

Among the 195 participants, 22.1% opted to prescribe antibiotics for the patient in the vignette, while 77.9% opted not to prescribe antibiotics.

### Patient characteristics influencing antibiotic prescription

#### Functional status

A statistically significant difference was observed across the three clinical vignettes, with the likelihood of antibiotic prescription increasing in correlation to the patient’s functional status. Among participants presented with vignette 3—describing a patient living at home and requiring only partial assistance with activities of daily living—still 62.1% chose not to prescribe antibiotics. In contrast, non-prescription rates were markedly higher for vignette 1 (91.2%) and vignette 2 (81.9%), both depicting patients residing in long-term care facilities with complete dependency.

Furthermore, antibiotics were less frequently perceived as representing therapeutic obstinacy in vignette 3. A significant difference was observed between vignettes 1 and 3 in this regard (*p* = 0.013).

#### Cognitive status

No significant difference in antibiotic prescribing was observed between the vignettes 1 and 2, which depicted a patient with advanced dementia (vignette 1) and a patient without cognitive impairment (vignette 2), who had the same level of physical functional dependence. However, we did observe a trend showing less antibiotic prescription for the patient with severe cognitive impairment, with 8.8% of participants opting to administer antibiotics for the patient in vignette 1, who had severe cognitive impairment, versus 18.1% for the patient in vignette 2, who did not, even though the difference was not statistically significant (*p* = 0.2).

#### Advance directives

The presence of advance directives was not significantly associated with antibiotic prescribing patterns. Nevertheless, the patient in vignette 2, who had advance directives stating she did not wish for therapeutic obstinacy, was less likely to receive antibiotics than the patient in vignette 3, who had no advance directives but better functional status (Table 2).

**Table 2:** Participants’ answers to the question “Would you prescribe antibiotics for the patient in the clinical vignette?”

Would you prescribe antibiotics for this patient?	Yes	**No**
Total	43 (22.1%)	152 (77.9%)
Vignette 1	5 (8.8%)	52 (91.2%)
Vignette 2	13 (18.1%)	59 (81.9%)
Vignette 3	25 (37.9%)	41 (62.1%)

### Physician characteristics influencing antibiotic prescription

Physicians practicing in French-speaking regions were significantly more likely to prescribe antibiotics compared to those in German- and Italian-speaking regions (33.7% vs. 13.8% and 11.1%, respectively). In contrast, prescribing behavior was not significantly associated with physician age, practice setting, or prior experience in palliative care.

### Reasons for prescribing antibiotics

The primary reason cited for prescribing antibiotics was the alleviation of dyspnea (51.3% of respondents). Other reported goals included treating the infection (38.5%), managing delirium (36.9%), reducing fever (19.0%), and prolonging life to allow death at home (16.9%). These results are summarized in Table 3.

**Table 3:** Reasons stated for prescribing antibiotics*

	**Reducing fever**	**Alleviating dyspnea**	**Managing delirium**	**Treating the infection**	**Prolonging life to allow death at home**	**Other**
All participants	37 (19.0%)	100 (51.3%)	72 (36.9%)	75 (38.5%)	33 (16.9%)	30 (15.4%)
Vignette 1	12 (21.1%)	28 (49.1%)	14 (24.6%)	21 (36.8%)	8 (14.0%)	9 (15.8%)
Vignette 2	8 (11.1%)	34 (47.2%)	31 (43.1%)	25 (34.7%)	12 (16.7%)	14 (19.4%)
Vignette 3	17 (25.8%)	38 (57.6%)	27 (40.9%)	29 (43.9%)	13 (19.7%)	7 (10.6%)

^*^Participants had the option to choose more than one response

Physicians were also asked to rate the influence of various clinical factors on their decision using a scale from 0 (no influence) to 10 (most influential). Among those who chose to prescribe antibiotics, the most influential factor was the presence of respiratory discomfort (median score: 7.7), followed by the presence of advance directives (6.6), new-onset fever (6.5), and the image of pneumonia on x-ray (6.1). For those who opted not to prescribe antibiotics, the most influential factor was the presence of multiple comorbidities (median score: 8.0), followed by the availability of alternative treatments for dyspnea relief (7.2), and the degree of functional dependency (6.8).

**Fig. 1:**
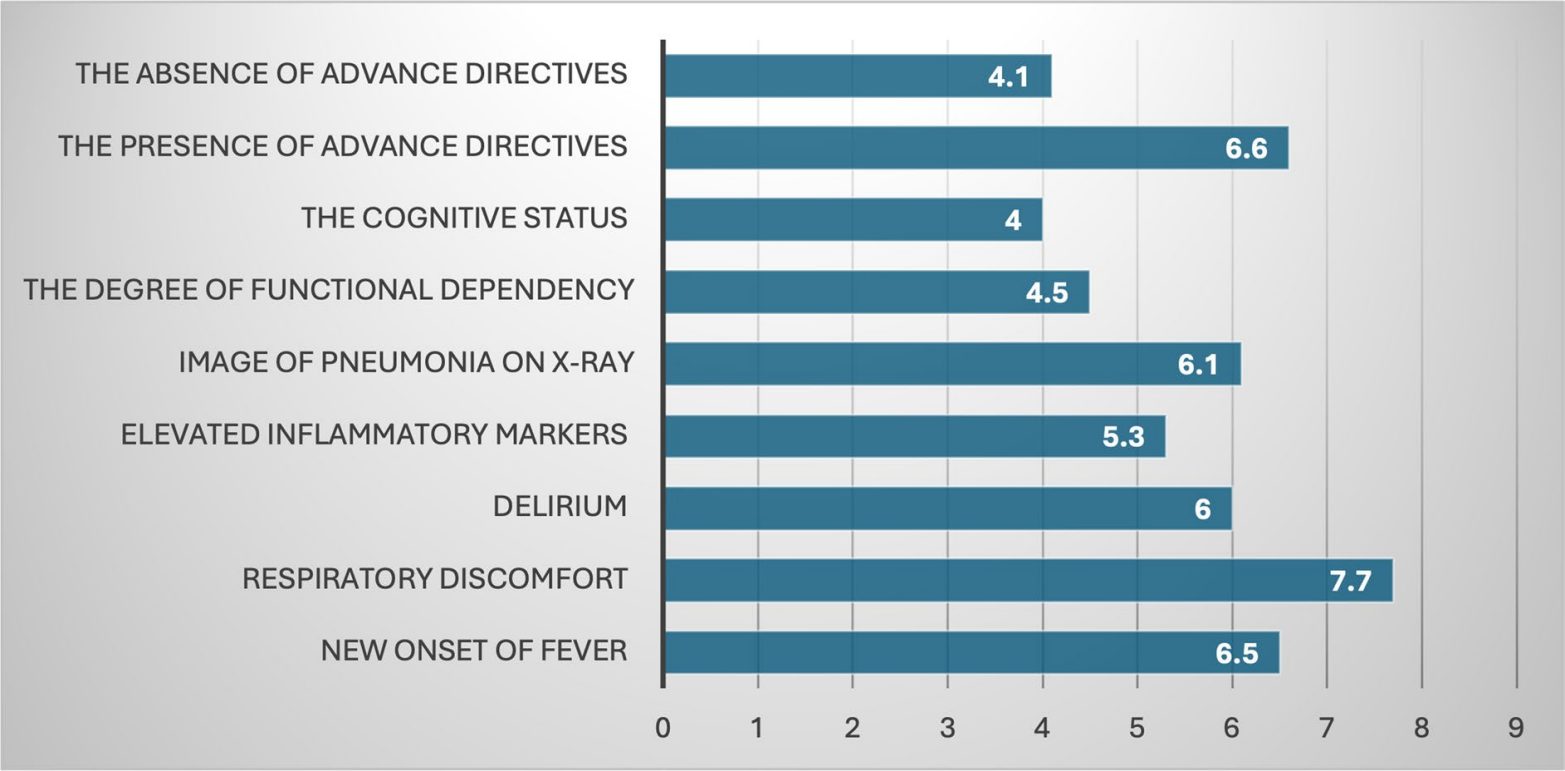
Participant rankings of the reasons for prescribing antibiotics* ^*^Results obtained on a scale of 0 to 10, with 0 = no influence at all and 10 = the most influential. Rankings are expressed in medians. Only participants who opted to prescribe antibiotics were asked this question

**Fig. 2:**
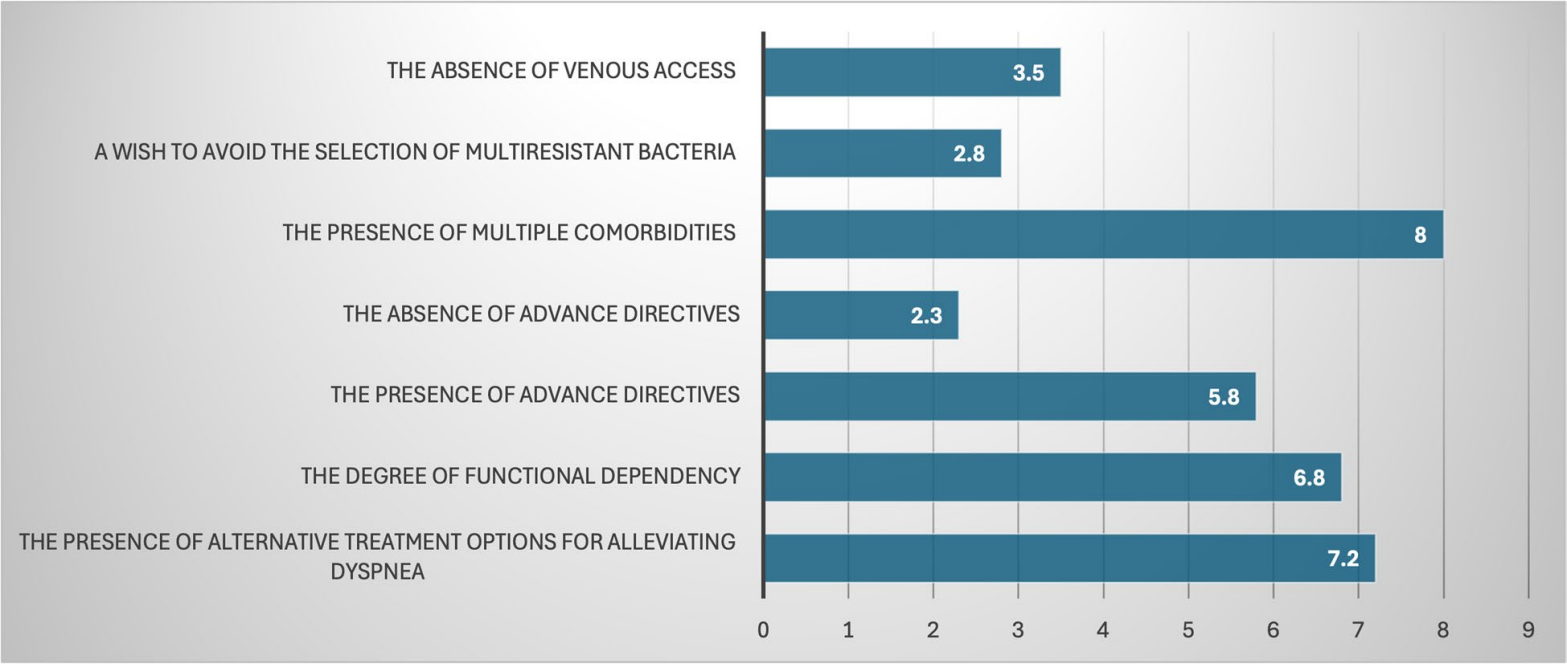
Participant rankings of the reasons for not prescribing antibiotics* ^*^Results on a scale of 0 to 10, with 0 = no influence at all and 10 = the most influential. Rankings are expressed in medians. Only participants who opted not to prescribe antibiotics were asked this question

The detailed results can be found in Fig. 1 and 2.

Most participants (76.4%) believed that withholding antibiotics could potentially shorten life, 77.9% did not consider antibiotics to improve patient comfort, and 61.5% stated that antibiotics had no role in end-of-life comfort care.

Nearly all participants (97.9%) reported that they would discuss their decision to initiate or withhold antibiotics with the patient’s family, and 90.8% indicated they would also involve the care team. Furthermore, 77.9% stated they would be willing to reconsider their decision if the patient’s family disagreed with their initial choice.

## Discussion

Our results highlight the importance of functional status as a factor associated with antibiotic prescription in end-of-life care. We also observed a marked ambivalence among physicians regarding the role of antibiotics in palliative care: while most respondents did not consider antibiotics to improve comfort or to have a legitimate role in end-of-life care, many still cited symptom relief—especially dyspnea—as a justification for their use.

The study also points to the importance of shared decision-making, with a large majority of physicians indicating they would involve both the care team and the patient’s family, and even adapt their decisions based on family preferences.

Finally, cultural and societal influences became apparent, with a significantly higher percentage of self-reported antibiotic prescription among physicians in the French-speaking regions of Switzerland compared with the German-speaking and Italian-speaking regions.

### Patient characteristics influencing antibiotic prescription

Some observational studies have previously demonstrated that physicians prescribe less antibiotics in end-of-life contexts for patients with severe dementia [8]. Some studies also suggest that functional decline may be useful as a prognostication marker in older adults [11, 15]. Finally, a surprisingly high number of patients with advance directives has been shown to receive antibiotics in their last two weeks of life [14]. However, a prospective cohort study by Manu et al. [25] reported that nursing home residents with comfort-oriented advance directives were less likely to receive antibiotics during hospitalization, even when these directives did not explicitly mention antibiotic use, while a study by Levin et al. showed that the absence of advance directives was associated with more antibiotic prescription at the end of life, as well as more colonization by multi-resistant organisms [24].

First, we found that functional status emerged as the leading factor influencing antibiotic prescription, with cognitive impairment showing a similar trend but without statistical significance. Measures of functional status, such as higher gait speed and better grip strength, have been associated with lower mortality in older adults in the community setting in several studies before [7, 21, 34]. Furthermore, hospitalized older patients who had better functional status in geriatric assessments had better survival outcomes [15].

A potential bias may have been introduced in the vignette featuring the patient with the least functional dependency, as this patient lived at home, contrary to the patient in the other two vignettes who had major functional dependency and lived in a care home. While it is possible that the living environment played a role, living at home primarily served as an indicator of greater independence within the context of the clinical vignette.

Among patients with cognitive impairment, several studies have similarly reported an association between lower functional scores and increased mortality, although findings remain inconsistent across the literature [28] These observations support using functional status as a key determinant in clinicians’ decision-making regarding antibiotic use at the end of life. Patients with higher functional capacity may indeed have a greater potential for short-term recovery, thereby influencing physicians to favor curative or life-prolonging interventions, including antibiotic therapy.

Second, we found no influence on antibiotic prescribing based on the presence or absence of dementia in clinical vignettes, independently of medical specialty (internal medicine versus geriatrics).

Interestingly, in a survey study by Crispim et al., the presence of severe dementia emerged as a primary factor limiting antibiotic prescription in end-of-life contexts, with 88 – 100% physicians choosing to prescribe antibiotics for terminally ill patients but only 45% physicians choosing to prescribe antibiotics if the patient also had advanced dementia [8]

This discrepancy between our study and the study by Crispim et al. may highlight broader challenges in recognizing dementia as a terminal condition and in appropriately limiting invasive interventions in this population, which has been described before [26].

Third, we reported no influence on antibiotic prescribing regarding the presence or absence of advance directives. This might be explained by the fact that advance directives do not consistently address the use of antibiotics in end-of-life scenarios. Interestingly, their presence ranked highly as an influencing factor, for both the prescribing and the non-prescribing group, with a median of 5.8 and 6.6 out of 10 respectively, but had finally, when making the decision, no clinical influence according to our survey.

### Physician characteristics influencing antibiotic prescription

We observed a difference in self-reported antibiotic prescribing across linguistic regions in Switzerland, with physicians in French-speaking areas more likely to prescribe antibiotics than their counterparts in German- and Italian-speaking regions. This finding aligns with broader European trends in antibiotic consumption. According to the 2023 epidemiological report by the European Centre for Disease Prevention and Control, total antibiotic use is significantly lower in Germany than in France, and a general North–South gradient is observed across Europe, with lower consumption in Northern countries and higher use in Southern ones [3]. These differences likely reflect underlying cultural influences on prescribing behavior, as previously documented. Indeed, studies suggest that countries with more traditional cultural values—common in Southern Europe—tend to have higher antibiotic use, whereas countries with more secular-rational values report lower use [27]. Furthermore, cultural orientations that prioritize survival have been associated with higher antibiotic consumption compared to cultures that emphasize quality of life and individual autonomy [10].

Possibly, these linguistic and cultural variations may be specific to antibiotic use rather than to end-of-life decision-making more broadly. A recent Swiss survey on beliefs about artificial nutrition at the end of life found no significant difference across linguistic regions [29], suggesting that regional variability may be more pronounced for infection-related interventions than for general palliative care practices.

In contrast to findings from a French study by Durand et al., we did not observe a difference in antibiotic prescribing across medical specialties [12]. In our sample, geriatricians, internists, and general practitioners reported similar prescribing patterns. These results may also be influenced by differences in medical training. In Switzerland, general practitioners and hospital-based internists share a common post-graduate training pathway, unlike in France, where these disciplines follow separate educational tracks, potentially contributing to the divergent findings between studies.

### Reasons for prescribing antibiotics

The presence of dyspnea was a major influencing factor of antibiotic prescription, which echoes the results of previous studies [12, 17]. Interestingly, alternative treatment options for alleviating dyspnea were highly ranked as an influencing factor for not prescribing antibiotics in our study, which may indicate that physicians are not convinced about the role of antibiotics in the treatment of dyspnea.

Physicians have a clear ambivalence regarding the role of antibiotics in palliative care. While 77.9% of participants stated they did not believe antibiotics improve comfort, and 61.5% considered that antibiotics have no place in end-of-life comfort care or symptom relief, dyspnea relief remained the most frequently cited therapeutic goal when antibiotics were prescribed. This apparent contradiction echoes findings from a similar French study by Durand et al., suggesting a disconnection between perceived clinical objectives and current evidence [12]. One possible explanation could be physician intervention bias leading to overtreatment by antibiotics in an effort to alleviate dyspnea. Indeed, the central role of dyspnea in the reasons for prescribing antibiotics reflects the psychological burden of dyspnea as a symptom, not only for the patients themselves, but also for caregivers and healthcare professionals. The difficulty of inaction in this context may account for some of the antibiotic prescription, even though other treatments, such as opiates and benzodiazepines, have been known to be more effective against dyspnea.

Indeed, the literature does not support a meaningful benefit of antibiotics for dyspnea in end-of-life care. Givens et al. reported higher discomfort scores among institutionalized patients with advanced dementia who received antibiotics compared to those who did not [18]. Similarly, Rosenberg et al. reported in a systematic review minimal symptom relief in terminally ill patients treated with antibiotics for pneumonia [32]. These findings raise concerns about the routine use of antibiotics as a comfort measure, particularly in the absence of strong supporting evidence.

Another noteworthy observation from this study is that 76.4% of respondents believed that withholding antibiotics could shorten life. While this perception may partly explain the tendency to continue antibiotic treatment near the end of life, it is not fully supported by current evidence. Several studies suggest that antibiotics may have a marginal effect on short-term survival, but this benefit is often limited in duration and does not translate into improved quality of life. Rather, these treatments may inadvertently prolong the dying process without meaningful clinical benefit [19, 35]. Notably, mortality from pneumonia in palliative care contexts remains high [30]—even when antibiotics are administered—raising critical questions about the appropriateness and potential futility of such interventions in end-of-life care [16].

### The role of nursing staff and family

The results of this study confirmed the important role of shared decision-making in antibiotic use at the end of life. Over 90% of participants indicated they would discuss the decision to initiate or withhold antibiotics with both the nursing team and the patient’s family. This proportion is markedly higher than reported in previous studies, where rates of physician-family discussions ranged from 7.9% to 46% [12, 33]. This discrepancy may reflect recall or social desirability bias inherent to self-reported survey data, or alternatively, a cultural context in Switzerland that emphasizes broader involvement of families and caregivers in end-of-life decisions, emphasizing the importance given to patients’ autonomy and informed consent. Given the survey’s low response rate, an alternative explanation may be selection bias, as physicians who chose to respond to the survey could be more interested in palliative care and potentially more sensitive to the involvement of nursing teams and families.

Notably, 77.9% of participants reported that they would be willing to revise their decision if the patient’s family disagreed with it. In light of the high rates of antibiotic prescribing at the end of life [26] this finding suggests that family preferences may strongly influence clinical choices. The major role of family and caregivers in the decision to prescribe antibiotics in end-of-life contexts has been described before [17, 23]. Improving antibiotic stewardship in this context will likely require greater investment in family and caregiver education about the risks and limited benefits of antibiotic therapy in terminal situations. Decision aids or structured guidance tools designed not only for physicians, but also for nursing staff and families could help support these complex conversations. One proposed approach is the use of time-limited antibiotic trials focused on symptom-oriented goals, accompanied by regular clinical reassessment to avoid unnecessary prolongation of treatment [16, 20].

### Study limitations

The primary limitation of this study was the low response rate and relatively small sample size, which may have limited the statistical power to detect associations between certain variables and antibiotic prescribing behavior. This may explain why no significant differences were observed in relation to key patient characteristics—such as the presence of dementia—or physician-related factors, including prior palliative care experience or medical specialty (e.g., geriatrics vs. internal medicine).

There is also a risk of selection bias given the low response rate, as physicians who were already interested in the topics of palliative care and antibiotic stewardship may have been more likely to participate.

Even though we tried to reflect national proportions in our initial sample, the response rate from French-speaking physicians was higher than the response rate from German-speaking physicians. This could be explained by closer similarities in the working culture and the way physician surveys are conducted in the French-speaking regions, as well as broader cultural and geographical closeness. As a result, selection bias may also be at play in the difference in antibiotic prescription observed between these regions, as the German-speaking participants may have been those particularly interested in antibiotic stewardship, and not entirely reflect the region’s antibiotic prescription practices.

Additionally, the clinical vignettes did not specify the severity of cognitive impairment using standardized tools such as the Clinical Dementia Rating (CDR) or the Montreal Cognitive Assessment (MoCA). This decision was made to maintain brevity and clarity in the questionnaire but may have limited the precision with which respondents interpreted the patient’s condition.

### Study strengths

The randomization of participants to one of three slightly modified clinical vignettes allowed for an objective assessment of the impact of functional status on antibiotic prescribing. The observed differences reached statistical significance, reinforcing the relevance of this clinical factor in end-of-life decision-making.

Furthermore, although the sample size was modest, it included a balanced representation of both hospital-based and ambulatory care physicians, enhancing the generalizability of the findings across different practice settings.

## Conclusions

The findings of this study highlight the need for enhanced communication tools and educational resources specifically designed for physicians, as well as for families and caregivers, to promote antibiotic prescribing that is medically appropriate and aligned with palliative care goals and patient values. Evaluating the clinical impact of such tools on real-world antibiotic prescribing at the end of life would be a valuable next step. Future research should further investigate the influence of cultural, institutional, and educational factors on end-of-life antibiotic prescribing practices. Large, multicenter studies incorporating real-world clinical data could help elucidate the role of antibiotics across diverse palliative care contexts and inform the development of targeted antibiotic stewardship interventions.

## Supplementary Information

Supplementary Material 1.

## Data Availability

The datasets used and/or analyzed during the current study are available from the corresponding author on reasonable request.
