# Supplementary material for: Antibiotic prescription for older patients in end-of-life care: a randomized survey among physicians in Switzerland
Source: BMC Geriatr. 2026 May 12;26:907. doi: 10.1186/s12877-026-07477-9 (PMC13334739; doi:10.1186/s12877-026-07477-9)
Supplement: Supplementary file 1 — Supplementary Material 1. [file 12877_2026_7477_MOESM1_ESM.docx]

**Full clinical vignette (and variations):**

The variations in vignette 1 are in **bold** characters, the variations in vignette 2 in *italics,* and the variations in vignette 3 are underscored.

Mrs R. is an 82-year-old female patient with a history of stroke, resulting in right-sided hemiparesis and aphasia, insulin-dependent type II diabetes mellitus, chronic lower limb arterial insufficiency with arterial ulcers, **and vascular cognitive impairment.**

She exhibits neurological dysphagia with recurrent aspiration pneumonia. The patient is currently receiving nutrition via percutaneous endoscopic gastrostomy and is strictly nil per os. ***Mrs. R. resides in a care facility and is completely dependent on third-party assistance for all basic daily activities. She is wheelchair-bound and requires assistance for mobility*** /

Mrs. R. lives at home with her husband and moves independently with the help of a walker. She is independent for most of her basic activities of daily living and is assisted by her husband for some of the instrumental activities of daily living.

The agreed-upon care plan, as discussed with her spouse and children, includes no cardiopulmonary resuscitation, no invasive ventilation or orotracheal intubation, and no non-invasive ventilation. **Mrs. R. has never completed advance directives** /

*In her updated advance directives from a few months ago, Mrs. R expresses that she does not wish to receive aggressive life-sustaining treatment. Her family’s priority is to ensure her comfort.*

Mrs. R. has been hospitalized for the fourth time this year due to a new episode of aspiration pneumonia. Three weeks ago, she was treated empirically with amoxicillin-clavulanate for a similar episode, and upon her current admission, she was started on piperacillin-tazobactam for seven days. Five days after the completion of piperacillin-tazobactam therapy, she developed a new febrile episode, accompanied by bronchial congestion and a worsening inflammatory response. Mrs. R. is experiencing significant discomfort and dyspnea at rest, with difficulty managing respiratory secretions. A chest radiograph reveals a new consolidation in the right lower lobe. A diagnosis of recurrent aspiration pneumonia is suspected.

Additionally, the patient is presenting with an altered mental status and has removed her only peripheral intravenous catheter. Her venous access is severely limited.

**Survey questions:**

The full list of questions can be found below, categorized by type of question (yes/no questions, rating scale questions, multiple choice questions, demographic questions):

Yes/no questions:

- Would you prescribe antibiotics for this patient?
- Do you consider antibiotics to be part of comfort care at the end of life?
- Do you believe that not initiating antibiotic treatment in the case of this patient could be considered negligence?”
- Do you believe that introducing antibiotic treatment in the case of this patient can be considered as therapeutic obstinacy?”
- Do you share the decision to initiate or not initiate antibiotic treatment in the case of this patient with the healthcare team?
- Do you share the decision to initiate or not initiate antibiotic treatment in the case of this patient with her family?
- If the patient’s family disagrees with your choice, will that change your decision?
- Do you believe that withholding antibiotic treatment in the case of this patient will shorten her life?
- Do you believe that initiating antibiotic treatment in the case of this patient will increase her comfort?”
- What is your level of certainty regarding this decision-making process?"

Rating scale questions:

If you start antibiotics, what is the order of the following factors that influenced your choice on a scale of 0 to 10? (0 = no influence, 10 = most important factor)” This question was only available for participants who replied that they would prescribe antibiotics.

- New onset of fever
- Respiratory discomfort Altered mental state
- Elevation of inflammatory markers
- Signs of pneumonia on radiograph
- Degree of functional dependence
- Cognitive status Presence of advance directives
- Absence of advance directives
- Other: specify

If you do not start antibiotics, what is the order of the following factors that influenced your choice on a scale of 0 to 10? (0 = no influence, 10 = most important factor)” This question was only available for participants who replied that they would not prescribe antibiotics.

- The presence of alternative treatments for managing the symptoms of fever/dyspnea/confusion
- The degree of functional dependence
- Cognitive status
- The presence of advance directives
- The absence of advance directives
- The presence of multiple comorbidities
- The desire to avoid the selection of resistant microorganisms
- The absence of venous access
- Other: specify

Multiple choice questions:

What would be the objectives of initiating a new antibiotic treatment in the case of this patient? (Multiple answers are possible)

- Lower the temperature
- Relieve dyspnea
- Relieve delirium symptoms
- Treat the lung infection
- Prolong the patient’s life so that she may pass away at her home

Demographic questions:

What is your age?

- = or < 30 years old
- 31-40 years old
- 41-50 years old
- 59-60 years old
- >60 years old

What is the language of your practice region?

- French
- German
- Italian

How many years since you obtained your medical degree?

- < 5 years
- 5 - 15 years
- >15 years

In which setting do you currently work?

- Ambulatory setting
- Hospital
- Both

If you work in a hospital setting, which department do you currently work in?

- General Internal Medicine
- Primary Care
- Geriatrics

Previous experience in specialized palliative care:

- None
- < 6 months
- 6 months-1 year
- >1 year

Do you hold a diploma/certificate in palliative care?

- Yes
- No

On average, how many times per month have you been faced with a decision to start or stop antibiotic therapy in an end-of-life context over the past year?

- None
- < 5 times
- 5-10 times
- >10 times

**Physician distribution and response rate in each linguistic region**

Total of German-speaking physicians contacted: 1514 (1183 GPs and 331 hospitalists) 62 % of the total sample

Total of German-speaking physicians who participated: 94 (48.2% of the total sample)

Response rate: 6%

Total of French-speaking physicians contacted: 812 (625 GPs and 187 hospitalists) 33 % of the total sample

Total of French-speaking physicians who participated: 83 (42.6% of the total sample

Response rate: 10%

Total of Italian-speaking physicians contacted: 129 (104 GPs and 25 hospitalists) 5 % of the total sample

Total of Italian-speaking physicians who participated: 18 (9.2% of the total sample)

Response rate: 14%

Total participants in all three regions: 2,455

Total participant rate: 7.9%
